# Evaluation of the Practicability of Biosynex Antigen Self-Test COVID-19 AG+ for the Detection of SARS-CoV-2 Nucleocapsid Protein from Self-Collected Nasal Mid-Turbinate Secretions in the General Public in France

**DOI:** 10.3390/diagnostics11122217

**Published:** 2021-11-27

**Authors:** Serge Tonen-Wolyec, Raphaël Dupont, Natalio Awaida, Salomon Batina-Agasa, Marie-Pierre Hayette, Laurent Bélec

**Affiliations:** 1 Ecole Doctorale Régionale D’Afrique Centrale en Infectiologie Tropicale, Franceville 876, Gabon; wolyec@gmail.com; 2 Faculty of Medicine and Pharmacy, University of Kisangani, Kisangani 2012, Congo; agasasalomon@gmail.com; 3 Laboratoire Paris XV, 75015 Paris, France; rjh.dupont@gmail.com (R.D.); famille.awaida@gmail.com (N.A.); 4 Department of Clinical Microbiology, University Hospital of Liege, 4000 Liege, Belgium; mphayette@chuliege.be; 5 Laboratoire de Virologie, Hôpital Européen Georges Pompidou, Assistance Publique-Hôpitaux de Paris, and Université of Paris, Sorbonne Paris Cité, 75015 Paris, France

**Keywords:** SARS-CoV-2, COVID-19, rapid diagnostic test, antigen, N nucleocapsid protein, nasal mid-turbinate secretions, self-test, general public, France

## Abstract

Due to their ease-of-use, lateral flow assay SARS-CoV-2 antigen-detecting rapid diagnostic tests could be suitable candidates for antigen-detecting rapid diagnostic self-test (Ag-RDST). We evaluated the practicability of the Ag-RDST BIOSYNEX Antigen Self-Test COVID-19 Ag+ (Biosynex Swiss SA, Freiburg, Switzerland), using self-collected nasal secretions from the turbinate medium (NMT), in 106 prospectively included adult volunteers living in Paris, France. The majority of the participants correctly understood the instructions for use (94.4%; 95% confidence interval (CI): 88.3–97.4), showing a great ability to perform the entire self-test procedure to obtain a valid and interpretable result (100%; 95% CI: 96.5–100), and demonstrated the ability to correctly interpret test results (96.2%; 95% CI: 94.2–97.5) with a high level of general satisfaction. About one in eight participants (# 15%) needed verbal help to perform or interpret the test, and only 3.8% of test results were misinterpreted. By reference to multiplex real-time RT-PCR, the Ag-RDST showed 90.9% and 100% sensitivity and specificity, respectively, and high agreement (98.1%), reliability (0.94), and accuracy (90.9%) to detect SARS-CoV-2 antigen. Taken together, our study demonstrates the high usability and accuracy of BIOSYNEX Antigen Self-Test COVID-19 Ag+ for supervised self-collected NMT sampling in an unselected adult population living in France.

## 1. Introduction

Coronaviruses are RNA viruses that cause common respiratory infections in humans, and they are an infectious agent responsible for mild respiratory tract infections [1]. Since December 2019, the coronavirus “SARS-CoV-2”, which causes severe bilateral pneumonia, has spread throughout the world, starting in Wuhan, China. The world continues to face the 2019 coronavirus pandemic (COVID-19). By October 2021, the World Health Organization (WHO) had noted more than 200,000,000 confirmed cases of COVID-19 infection and nearly 5,000,000 deaths due to this virus.

Testing is one of the principal strategies to fight infectious diseases, as it is the gateway to prevention and treatment strategies. Furthermore, massive screening of people potentially infected with SARS-CoV-2 is essential to limit the spread of SARS-CoV-2 [2,3].

While currently recommended nucleic acid amplification tests, such as real-time reverse transcription-polymerase chain reaction assays, remain the gold-standard cornerstone for the diagnosis of SARS-CoV-2 infection [4,5], immunological methods can also be used to detect viral antigens [5,6,7]. Thus, antigen-detecting rapid diagnostic tests (Ag-RDT) enable new testing strategies for SARS-CoV-2 infection diagnosis and control due to their short turn-around time and ease-of-use [6,8,9]. However, most SARS-CoV-2 Ag-RDTs rely on nasopharyngeal sampling, and their use is hampered by the necessity to deploy qualified healthcare workers and protective equipment to collect nasopharyngeal secretions, perform the tests and interpret the test results. In addition, a nasopharyngeal swab is often considered uncomfortable, which limits the ability of frequently performing screening tests from this type of nasopharyngeal sampling [10].

Alternative sampling methods, such as nasal mid-turbinate (NMT) sampling (swab inserted about 2–3 cm into nostril parallel to the palate until resistance is met at turbinates), is well tolerated and can be performed by the person himself either unsupervised at home or supervised on-site [10,11,12,13]. NMT sampling was equivalent to nasopharyngeal sampling for a WHO-listed SARS-CoV-2 Ag-RDT, which lays the foundation for its use as a self-sampling technique under supervision [12,13,14,15]. Widespread use of self-testing for SARS-CoV-2 infection could help improve the control of the spread of the SARS-CoV-2 epidemic [16,17,18,19,20,21]. Previous experience with HIV self-testing showed that a self-test can be performed perfectly well by a layperson and gives reliable and accurate results, and that HIV self-testing is considered as an acceptable and useful tool to improve HIV testing coverage worldwide to improve HIV epidemic control [22,23]. Lateral flow tests for the SARS-CoV-2 antigen are appropriate for use as an antigen-detecting rapid diagnosis self-test (Ag-RDST) because of their low cost, ease of use, and prompt result [12,14,24].

Recent reports have established the achievability of NMT self-sampling under supervision [12,14]. Data on the clinical performance of self-testing with Ag-RDT are, however, limited to comparisons with quantitative real-time RT-PCR (rtRT-PCR) detection of SARS-CoV-2 RNA [25,26].

This study aimed to evaluate the usability and clinical performance in the field of the Ag-RDST BIOSYNEX Antigen Self-Test COVID-19 Ag+ (Biosynex Swiss SA, Freiburg, Switzerland; reference 859271) for COVID-19 antigenic testing from self-collected NMT secretions in adults living in the Paris region during the third wave of the COVID-19 epidemic in France. For the first time to our knowledge, the usability of this novel Ag-RDST was evaluated according to a systematic approach based on our previous experience in assessing HIV and SARS-CoV-2 self-testing using capillary blood samples [27,28,29,30].

## 2. Materials and Methods

### 2.1. SARS-CoV-2 Antigen-Detecting Rapid Diagnostic Test for Self-Testing

The CE IVD-labeled Ag-RDST BIOSYNEX Antigen Self-Test COVID-19 Ag+ (Biosynex Swiss SA) is a rapid qualitative membrane-based immunochromatographic test that uses highly sensitive monoclonal antibodies to detect the SARS-CoV-2 N nucleocapsid protein in nasopharyngeal secretions sample. The Ag-RDST BIOSYNEX Antigen Self-Test COVID-19 Ag+ is packaged for individual use with seven components placed in an aluminum pouch containing the test cassette, a diluent tube pre-filled with buffers (sample, reagent, and absorbent), a dropped teat, swab (Jiangsu Changfeng Medical Industry Co., Yangzhou, China; CE marking: CE0197), and a sachet of desiccant (to be thrown away). The box has a cut-out hole to place the pre-filled diluent tube. The test includes a reaction membrane containing colloidal gold particles conjugated to monoclonal antibodies and secondary antibodies directed against the N protein of SARS-CoV-2 that are placed inside a plastic cassette.

The self-test was performed according to manufacturer instruction. In brief, the top of the pouch is removed following the indent, in order to remove the cassette. The seal is removed from the pre-filled diluent tube, which is placed in the cut-out hole in the box. Then, the swab is removed from its packaging. The swab should be vertically inserted into a nostril, until it starts to resist (at approximately 3 cm deep). The swab is rolled 5 times along the inner walls of each nostril, to ensure that the nasal secretions and cells are collected. Afterwards, the swab is inserted into the pre-filled diluent tube and is rotated for at least 10 s whilst the end of the swab is pressed against the bottom of the extraction tube. It should be mentioned that as much secretion as possible should be collected from the swab by “squeezing it out” using the edge of the tube, by pressing the swab against the edge of the tube several times. Then, the swab is removed from the diluent tube, which is closed by the dropper teat. Using the dropper, 4 drops of solution are slowly deposited into well “S”.

If SARS-CoV-2 antigens are present in the sample, a red line appears in the test line area (T). The result is negative when no red line appears in the test line area. An internal procedural control is assured by a red line appearing in the control line area (C). Visual interpretation of results is performed 10 min after. The results should not be read after 15 min.

The test must be performed at room temperature (15–30 °C) and in an environment free from excessive humidity, within 1 h after opening.

The analytical performances of the self-test Ag-RDT BIOSYNEX Antigen Self-Test COVID-19 Ag+ are given in the instructions for use (Figure S1), with a sensitivity of 96.0% (95% confidence intervals (CI): 90.9–98.7%) and a specificity of 99.2% (95%CI: 95.4–99.9%).

The simplified instructions for use of the BIOSYNEX Antigen Self-Test COVID-19 Ag+ were comprised of an easy-to-read leaflet in French and other languages (English, German, Dutch, and Italian) in A3 format color printing. The printable instruction for use in French is depicted as supporting information (Figure S1). The online instruction in the video for use was available online from YouTube (in French: https://www.youtube.com/watch?v=R9fxagzoFSs&list=PLQqQgOWEz169GgQECgZIMW04qcvnpTC1-&index=1 (accessed on 3 November 2021); in English: https://www.youtube.com/watch?v=MVDFQZJZTig&list=PLQqQgOWEz169GgQECgZIMW04qcvnpTC1-&index=3 (accessed on 3 November 2021)).

The BIOSYNEX Antigen Self-Test COVID-19 Ag+ has obtained the CE-IVD mark in 2021, and is sold in many European countries (Austria, Belgium, Denmark, France, Germany, Italy, Sweden, and The Netherlands) (around 800,000 tests per month). Biosynex Swiss SA (Freiburg, Switzerland) is the Own Brand Labeler which has designed this self-test, and ensures all the packaging, and national quality controls of manufacturing.

### 2.2. Practicability Evaluation

The practicability evaluation of the BIOSYNEX Antigen Self-Test COVID-19 Ag+ is a prospective study performed during the third wave of the COVID-19 epidemic between April and May 2021 by recruitment of adult volunteers aged ≥18 years receiving testing for SARS-CoV-2, at the Laboratoire Paris XV, Paris, France, consisting of face-to-face, paper-based, semi-structured, and self-administrated questionnaires.

### 2.3. Study Design and Recruitment of Participants

In France, the molecular test for SARS-CoV-2 RNA detection is carried out voluntarily or on medical prescription and the test is fully covered by the health insurance [31,32,33]. The study site offered SARS-CoV-2 testing to anyone in the community who wanted testing by conventional molecular diagnosis with a nasopharyngeal swab for suspected COVID-19, for travel, as part of a preoperative assessment, exposure as a contact case of an individual infected with SARS-CoV-2, or control of past infection with SARS-CoV-2 within 30 days. A questionnaire capturing demographic information (sex and age), reasons for testing, and current and past–14-day symptoms (headache, fatigue, fever, or upper or lower respiratory symptoms) for symptomatic patients was administered to all participants. Asymptomatic individuals were defined as those not reporting any of these symptoms in the past month.

For rtRT-PCR, a health care professional collected nasopharyngeal secretions in one nostril, using a flocked swab. The nasopharyngeal swab sample was discharged in 1000 μL of physiological serum (NaCl 0.9%), and stored at +4 °C before processing. The laboratory gives the diagnosis of SARS-CoV-2 RNA detection within 24 h by SMS, internet or in the form of a paper report.

After the nasopharyngeal swab sampling for SARS-CoV-2 RNA molecular testing, the objectives of the practicability and analytical evaluation of the BIOSYNEX Antigen Self-Test COVID-19 Ag+ were explained to the participants. Eligible participants were of an age ≥18 years, wanted to use on-site for the first time for a self-test Ag-RDT using nasal secretions for SRAS-CoV-2 detection, were capable to speak and read in French, and gave their informed consent to participate in the study. Anyone already trained in the use of rapid diagnostic tests, such as doctors, nurses and technicians or biologists, were excluded.

### 2.4. Practicability Study Outcomes

**Figure 1:**
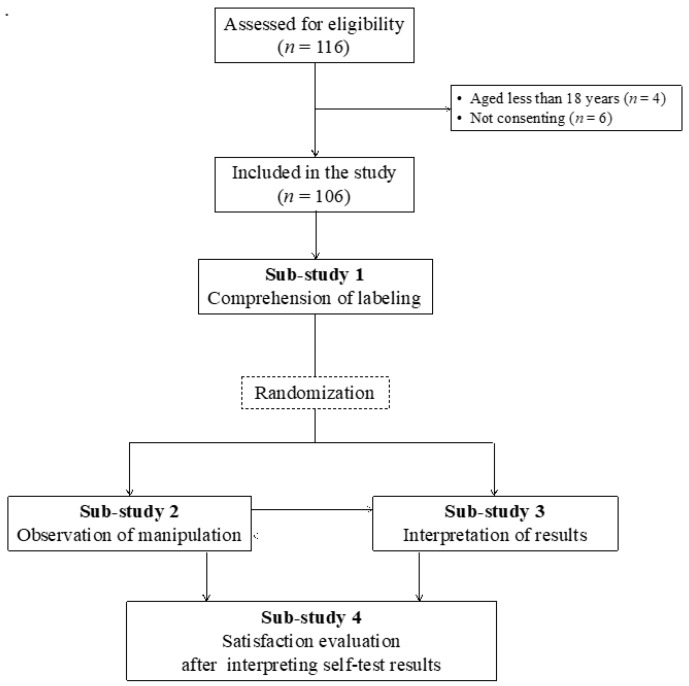
Recruitment of study participants.

The practicability evaluation was divided into four sub-studies carried out by trained health care professionals, based on previously acquired experience from WHO recommendations for evaluating the practicability of HIV and COVID-19 self-tests [27,28,29,30]. While all participants were included in the study concerning the understanding of labeling (sub-study 1), they were randomized into two groups for studies concerning manipulation of the test (sub-study 2) and evaluating the interpretation of COVID-19 self-test results (sub-study 3), using block randomization of 4. A cross-over was then made, allowing the participants of sub-study 2 to participate in sub-study 3 and vice versa. Finally, all participants were asked to fill the satisfaction questionnaires (sub-study 4) (Figure 1).

### 2.5. Data Collection and Procedures

Paper-based, structured questionnaires were completed by each participant in order to collect their socio-demographic characteristics, their medical history, to assess their understanding of the instructions for use, and to obtain their opinions or levels of satisfaction about the practicability of the BIOSYNEX Antigen Self-Test COVID-19 Ag+. All data relating to the observation of manipulation and the interpretation of test results were recorded on the standardized sheets by the observers.

#### 2.5.1. Sub-Study 1

Comprehension of labeling. After receiving a brief explanation of the objectives and conduct of the study, the participants were asked to give their oral informed consent. In a private setting, the participants had the choice between a paper-based instruction for use and a video-based instruction for use (Biosynex autotest antigénique COVID-19 Ag+; available at: https://youtu.be/R9fxagzoFSs (Accessed on: 3 November 2021)), which they were asked to read or watch and understand independently. After declaring that the instructions for use were understood, participants were asked to complete a questionnaire to assess their understanding. This questionnaire included 11 questions concerning the key information of the instruction for use with closed answers (true, false, or do not know). That key information included the manipulation of the kit, the interpretation of test results, and the consequence of the test results. The participants who correctly answered all 11 questions were considered to have correctly understood the instructions for use.

After this survey, participants were randomized into two groups for evaluation on performing the self-test and the interpretation of test results, using a sealed randomization envelope.

#### 2.5.2. Sub-Study 2

Observation of manipulation. In a private setting supervised by an observer, each participant received a box containing the BIOSYNEX Antigen Self-Test COVID-19 Ag+. Participants were then asked to carry out the self-test by themselves in front of a trained observer. The observer was responsible for recording the respect or not of each step, appeal for verbal assistance (mimicking telephone support), difficulty, and errors on a standardized sheet. The successful performance of the SARS-CoV-2 antigenic self-test was conditioned by the presence of the control band on the test strip, and the results of the Ag-RDT were read and recorded independently by participants and observers.

#### 2.5.3. Sub-Study 3

**Figure 2:**
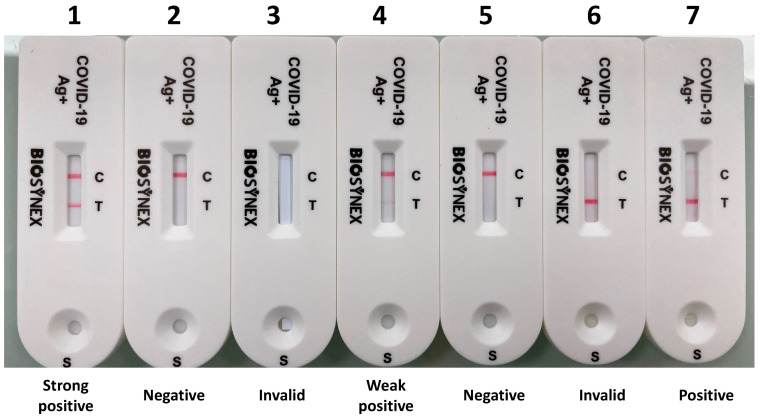
Panel of 7 BIOSYNEX Antigen Self-Test COVID-19 Ag+ (Biosynex Swiss SA) cassettes, including 3 positive tests (strong positive: n°1; weak positive: n°4; and positive: n°7), 2 negative tests (n°2 and n°5) and 2 invalid tests (n°3 and n°6). Each participant drew and interpreted five of the seven tests and the results were recorded by the observer.

Interpretation of test results. In a private setting supervised by an observer, seven standardized test results including three positive tests (positive, weak positive, and strong positive), two negative tests, and two invalid tests were proposed to the participants for interpretation after successive random selection of five tests (Figure 2).

#### 2.5.4. Sub-Study 4

Satisfaction questionnaire. Finally, the participants fulfilled the satisfaction questionnaire concerning their experiences with the BIOSYNEX Antigen Self-Test COVID-19 Ag+.

### 2.6. Laboratory Procedure

Nucleic acid extraction was performed from 300 μL thawed aliquots of the eluted nasopharyngeal swab samples using automated nucleic acid extraction EX3600 extractor (Liferiver & Shanghai ZJ Bio-Tech Co., Shanghai, China) with the Liferiver^®^ Viral RNA Extraction kit (Liferiver & Shanghai ZJ Bio-Tech Co.; reference NE-0044). The BIOSYNEX AmpliQuick^®^ SARS-CoV-2 (Biosynex Swiss SA) is a multiplex rtRT-PCR assay for the detection of SARS-CoV-2 RNA, according to the manufacturer’s instructions. The kit is composed of a 96-well microplate pre-filled with the master mix containing dNTPs, MgCl2, fluorescent primers and probes, Taq polymerase and reverse transcriptase enzymes, and reaction buffer. The assay can simultaneously detect 2 coronavirus target genes, including the SARS-like (including SARS-CoV-2, SARS-CoV, and bat SARS-like coronavirus) conserved region of envelope protein gene (E), and RNA-dependent RNA polymerase gene (ORF1ab of RdRP gene), providing individual cycle threshold (Ct) values for each target gene. This assay was performed on the CFX96™ Real-Time PCR Detection System (Bio-Rad Laboratories, Hercules, CA, USA), according to the manufacturer’s instructions.

The results of SARS-CoV-2 RNA detection obtained by the multiplex rtRT-PCR were considered as the reference.

### 2.7. Statistical Analysis

The minimum sample size was calculated using Fisher’s test with the assumption of 80% expected frequency corresponding to the success of practicability (sub-studies 1, 2, and 3), 5%-alpha risk and 20%-beta risk. Thus, the minimum sample size was estimated to be 105 participants.

All data (Excel file) were analyzed using SPSS 20.0 (Chicago, IL). Descriptive statistical analyses were carried out using mean, standard deviation, median, interquartile range, frequency, and percent. The 95% confidence interval (CI) was calculated using Wilson score bounds [34]. The results of SARS-CoV-2 RNA detection by the multiplex rtRT-PCR were used as the reference standard to estimate the sensitivity and specificity of the study self-test Ag-RDT, with corresponding 95% CI. The percent agreement and concordance between study self-test and reference test corresponded to the observed proportion of identical results between Ag-RDT compared to rtRT-PCR detection. The reliability between the study self-test Ag-RDT and the multiplex molecular detection of SARS-CoV-2 RNA was estimated by Cohen’s κ coefficient [35], and the degree of agreement was determined as ranked by Landlis and Koch [36]. Youden’s J index (J = sensitivity + specificity − 1) was used to estimate the accuracy of the study self-test [37].

### 2.8. Ethics Statement

The study is part of the continuous laboratory quality improvement program for the diagnosis of SARS-CoV-2 infection, in accordance with European guidelines on the accreditation of medical biology laboratories [38]. The dataset was completely anonymous. In addition, all participants fulfilled the recommendations of the Haute Autorité de Santé (Saint-Denis, France) of 21 April 2021, on the use of self-tests for SARS-CoV-2 detection, i.e., in asymptomatic persons over 15 years of age, within the framework of large-scale iterative screening campaigns, as well as in the private setting for individuals [39].

## 3. Results

### 3.1. Study Population

A total of 116 individuals were assessed for eligibility, but 10 were excluded because they were less than 18 years old (n = 4), and not consenting (*n* = 6) (Figure 1). Finally, 106 were successfully enrolled in all practicability sub-studies and clinical performance evaluation.

The demographic characteristics and medical history of study participants are shown in Table 1. Overall, 68 (64.2%) were female. The median age was 40 years, and the majority of participants were aged between 21 and 59 years. The majority (83.9%) of participants possessed at least a college or higher education level. The majority (87.7%) of them possessed no previous experience of self-testing for diseases other than COVID-19.3.1. Main reasons for testing were air travel (*n* = 41; 38.7%), case-contact exposure of an individual infected with SARS-CoV-2 (*n* = 10; 9.4%), suspected COVID-19 (*n* = 30; 28.3%), pre-operative assessment (*n* = 18; 17.0%), and control of SARS-CoV-2 infection in the 30 days preceding (*n* = 7; 6.6%). At the time of testing, the majority (71.7%) of participants had reported no symptoms of COVID-19 in the past month, including all contact-cases from SARS-CoV-2-infected individuals. Around one-third of participants reported at least one COVID-19-compatible symptom. Among symptomatic patients, the median time of symptom duration before sampling was 4 days (range, 0–7 days).

### 3.2. Practicability Evaluation

#### 3.2.1. Sub-Study 1

This sub-study evaluated the ability of the 106 study participants to understand the instructions for use of the BIOSYNEX Antigen Self-Test COVID-19 Ag+. The majority (*n* = 76; 71.7%) of participants preferred to use the paper-based instructions whereas 30 (28.3%) participants used the video-based instructions. The analytical results of the evaluation questionnaire are shown in Table 2. Overall, 100 (94.4%; 95% CI: 88.3–97.4) participants correctly understood the instructions for use, thus correctly answering all 11 questions. The labeling index for participant understanding, which measured the mean of the correct answers for each question was 96.8% (95% CI: 91.5–98.8). The question concerning the necessity that the nasal swab must touch the inner walls of the nostrils and that concerning the lack of control band showed the highest rate (5.6% and 4.6%, respectively) of incorrect responses.

#### 3.2.2. Sub-Study 2

This sub-study evaluated the ability of participants to use the study self-test Ag-RDT BIOSYNEX Antigen Self-Test COVID-19 Ag+ in a supervised setting. Overall, all participants (100%; 95% CI: 96.5–100) performed the self-test and succeeded in obtaining a valid test result with an overall usability index of 99.1% (95% CI: 94.9–99.9) (Table 3). A total of ninety-two (86.8%; 95% CI: 79.1–92.0) participants correctly used the self-test without any difficulties, errors, or help, whereas 14 (13.2%; 95% CI: 8.0–20.9) had asked for verbal help. The introduction of the nasal swab in both nostrils, and the necessity to turn the swab 6 times in the diluent tube were the steps requiring the most frequent verbal help in 7.5%, and 5.7%, respectively (Table 3). Interestingly, all participants (*n* = 30; 28.3%) using the video instructions performed the self-test easily (usability index of 100%) without any difficulties, errors, or help. Overall, the mean time of antigenic self-test performance (since the opening of the box until the migration step) was 8.1 (SD: 1.3) minutes.

#### 3.2.3. Sub-Study 3

**Figure 3:**
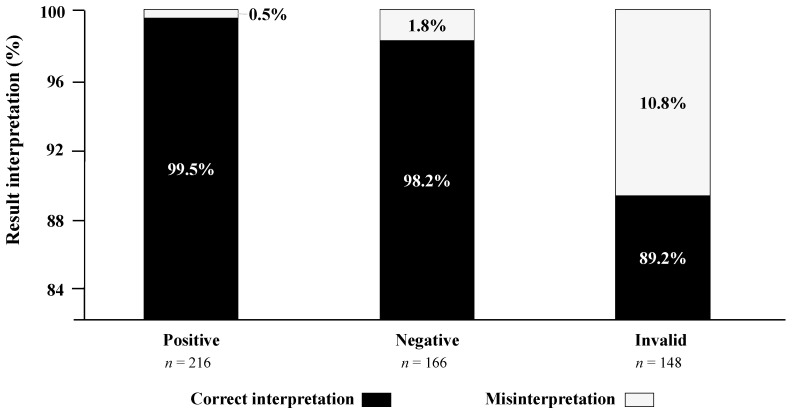
Stacked columns showing the ability of participants to read and interpret (correctly or incorrectly) the 530 results of the BIOSYNEX Antigen Self-Test COVID-19 Ag+ (Biosynex Swiss SA) obtained from successive random selection of a panel of 7 standardized tests, including three positive, two negative, and two invalid test results.

This sub-study evaluated the ability of participants to read and interpret the COVID-19 antigenic self-test results after successive random selection of five tests from a panel of seven standardized tests. The results are depicted in Figure 3. Overall, 530 standardized tests were read and interpreted by the 106 participants, including 216 positive, 166 negative, and 148 invalid test results. A total of 510 (96.2%; 95% CI: 94.2–97.5) tests were correctly interpreted, whereas 20 (3.8%; 95% CI: 2.8–5.8) tests were misinterpreted.

Misinterpretation occurred in 0.5% (*n* = 1) of positive tests (result weakly positive falsely interpreted as negative), in 1.8% (*n* = 3) of negative tests (result negative falsely interpreted as positive) and in 10.8% (*n* = 16) of invalid tests falsely interpreted as positive. Cohen’s κ coefficient between the results of reading by participants and the expected results was 0.95, demonstrating an excellent concordance.

#### 3.2.4. Sub-Study 4

This sub-study assessed the post-test satisfaction of participants concerning the instructions for use (sub-study 1), performing the COVID-19 antigenic self-test (sub-study 2), and the interpretation of test results (sub-study 3). The results of the questionnaire are shown in Table 4. The majority of items were considered very easy or easy. The use of the video on YouTube to help with the realization of the self-test was appreciated as very easy by 80% of participants, having chosen this option. Regarding their ability to overcome the difficulties encountered during the COVID-19 self-test, all (100%) participants responded by saying that it was easy (94.4% very easy; 5.6% rather easy). The majority of participants would repeat such antigenic self-test if necessary (78.3%) and would recommend it to someone else (93.4%).

### 3.3. Analytical Performance by Lay Users

Analytical performance by lay users. Using rtRT-PCR as the standard, two false-negative antigenic self-test results occurred, among specimens from asymptomatic (*n* = 1) or symptomatic (*n* = 1) participants. No false-positive antigenic self-test result was observed. The mean level of viral excretion assessed by the mean Ct values of E and RdRP genes by the reference rtRT-PCR was higher in symptomatic than asymptomatic participants (23.1 ± 2.8 versus 28.2 ± 2.7 arbitrary units; *p* = 0.021 by Mann–Whitney U test). Overall, the Ag-RDT BIOSYNEX Antigen Self-Test COVID-19 Ag+ showed high sensitivity and specificity of 90.9% and 100%, respectively, and high or almost perfect agreement (98.1%), reliability (0.94), and accuracy (90.9%) to detect SARS-CoV-2 (Table 5).

## 4. Discussion

We herein report on our recent experience during the third COVID-19 epidemic peak period in France of the usability and clinical performance in the field of a novel Ag-RDST for COVID-19 antigenic testing from self-collected NMT secretions in adult volunteers living in the Paris region. The majority of participants were able to correctly understand the instructions for use of the self-test, to obtain a reliable result and to interpret the final test results. About one out of eight (#15%) participants needed verbal help, and only 3.8% of test results were misinterpreted. Taken together, our study demonstrates the feasibility and accuracy of COVID-19 self-testing from self-collected NMT sampling in an unselected adult population in a supervised setting. These observations lay the foundations for the potential large-scale use of COVID-19 Ag-RDST in lay adults, at least Europeans, to complete the arsenal of available diagnostic tests for SARS-CoV-2.

### 4.1. Practicability Evaluation

The overall usability of the BIOSYNEX Antigen Self-Test COVID-19 Ag+ was evaluated by four successive sub-studies that allow a good analysis of all the steps of the test realization, from the understanding of the instructions of use with the identification of the test components, to the realization of the Ag-RDST and the final interpretation of the results. The satisfaction sub-study is also essential to assess the acceptability of the self-test, which will necessarily have to be repeated in an epidemic context.

The sub-study 1 tested whether the instructions for use can be read and understood by all participants. Our findings showed that 94.4% of participants correctly answered all 11 questions, indicating a generally correct understanding of the key messages delivered by the instructions for use of the BIOSYNEX Antigen Self-Test COVID-19 Ag+, with an overall rate of good responses of 96.8%. These satisfactory results may be explained in part by a sufficient education level of the majority of study participants. Indeed, previous experience with HIV self-diagnosis has shown that an insufficient level of education is a great challenge to understanding the instructions for use [22,40]. Although systematic reviews and meta-analysis have shown that HIV self-diagnosis can be successfully conducted by untrained users without help [22], our observations underscore the need to supplement the traditional paper instructions with other educational tools such as a short video, which was preferred by 28.3% of study participants for better understanding of the instructions for use. These findings are reminiscent of previous WHO recommendations for HIV self-testing, that all self-testers should have the option of accessing assistance via telephone, the internet, or additional instructions such as videos, animations, or diagrams [41]. The difficulty in explaining that the swab must touch the bottom of the inner wall of the nostril for correct NMT sampling must be emphasized. This difficulty, encountered in 5.6% of participants, could be the cause of false negative results.

In sub-study 2, all study participants carried out the COVID-19 Ag-RDST and succeeded in obtaining a valid test result with an overall usability index estimated at 99.1%. The time of realization of the test was particularly short (8.1 min). Some difficulty in the correct introduction of the nasal swab in both nostrils and the necessity to turn the swab 6 times in the diluent tube were the principal reported concerns encountered and were the most common reason for oral help. All participants using the video instructions performed the self-test easily. These features underscore the importance of video instructions, when available. The use of a hotline could also provide direct remote assistance.

The ability to correctly read and interpret the results of the self-test is considered a sensitive step in self-testing [42]. This refers not only to the visual ability related to good visual acuity when reading and interpreting the results, but also to the intensity of the bands to be read on the strip. In our series, the rate (96.2%) of correct interpretation of COVID-19 Ag-RDST results was high, as usually observed for example with HIV self-test using a similar cassette [28,29,30]. In one case, the misinterpreted test results concerned a weak positive band, read as negative. This difficulty in reading some weak positive bands and definitively interpreting the test results can occur in non-professional users as well as in users trained in professional testing [43]. The misreading of a weakly positive strip is obviously restrictive because the subject falsely believes he is negative. However, the instructions for use clearly state that a negative test does not mean that the Ag-RDST is not contagious. The misreading of invalid tests interpreted as positive should in principle be corrected by further COVID-19 molecular testing, which must be systematic in case of positivity, as indicated in the instructions for use.

The post-test answers to the satisfaction questionnaire concerning the instructions for use (sub-study 1), performing the self-test (sub-study 2), and the interpretation of the results (sub-study 3), showed that the large majority of the COVID-19 self-testing steps were considered easy or very easy by the majority of participants, as previously reported for HIV self-testing using a similar rapid test cassette [28,30]. Watching a video before the test was particularly appreciated. The willingness to repeat the use of the study Ag-RDST when necessary is important for widespread use, because the Ag-RDT for SARS-CoV-2 detection must be repeated to identify infectious individuals, or for private personal use so that it becomes routine.

Taken together, our observations highlight in adult profanes the usability of the BIOSYNEX Antigen Self-Test COVID-19 Ag+ using painless NMT self-sampling as a novel approach to assess SARS-CoV-2 infection by using Ag-RDT test and self-interpretation of the results in a supervised setting. The feasibility of nasal swabs self-collection in the community was previously emphasized for the diagnosis of respiratory virus infection, such as influenza A and B and other common respiratory viruses, including common coronaviruses [44,45], and these swabs appear to be both easy to perform [42,43,44,46,47,48] and well accepted [46,48]. Self-collection of nasal secretions for respiratory viruses offers potential significant benefits by reducing the requirement for personal protective equipment, limiting exposure of patients and staff to infection, increased convenience and access for patients and timeliness of a sample receipt [45,48,49]. In the present series, the handling of the test itself did not pose any particular problem, as previously demonstrated for other self-tests with similar cassettes [28,29,30,50]. NMT self-sampling was acceptable and achievable over a range of education levels, and the majority of participants had a preference for this method over health care collection, likely because of the ability of patients to control the comfort level of nasal collection better than a trained collector can, as has previously been reported for other respiratory viruses [46,47,48]. Finally, the interpretation of the two bands on the strip of the study Ag-RDST was generally correct, as previously shown for rapid diagnostic tests of comparable forma in lay users from general adult population [28,29].

### 4.2. Analytical Performance by Lay Users of the BIOSYNEX Antigen Self-Test COVID-19 Ag+

The analytical performance of the study Ag-RDST was evaluated by reference to multiplex rtRT-PCR for SARS-CoV-2 RNA detection as gold standard in a real-life community setting. In this evaluation, the sensitivity of the BIOSYNEX Antigen Self-Test COVID-19 Ag+ was lower among specimens from asymptomatic persons (83.3%) than among specimens from symptomatic persons (93.8%). Specificity (>99.0%) was high in specimens from both asymptomatic and symptomatic groups. The prevalence of having SARS-CoV-2 rtRT-PCR positive test results in this population was relatively high (20.7% overall; 7.9% for asymptomatic participants and 53.3% for symptomatic participants). The high viral load of SARS-CoV-2 across the upper and lower respiratory tracts, including nasal sites [41,47,48,51] and sensitive molecular techniques may explain the equivalent sensitivity of nasal self-collection to health care collection samples in COVID-19 patients.

The study Ag-RDST fulfilled the current WHO’s recommendations for a screening Ag-RTD stating that, at minimum, Ag-RDTs would need to correctly identify significantly more cases than they would miss (sensitivity ≥80%) and would have very high specificity (≥97–100%) [52]. Furthermore, analytical performances must be of a comparable order, as those participants in our study of Ag-RDT were previously reported for some Ag-RDTs in lateral flow immunoassay format [9,53,54,55,56,57,58,59,60,61], whereas several studies have reported much lower sensitivity levels contrasting with always high specificity [6,62,63,64,65]. In addition, the Ag-RDST BIOSYNEX Antigen Self-Test COVID-19 Ag+ fulfilled the current recommendations of the Haute Autorité de Santé (Saint-Denis, France) for a screening Ag-RTD, stating that, at minimum, Ag-RDTs would need to correctly identify significantly more cases than they would miss in symptomatic patients (sensitivity ≥80%) as well as in asymptomatic individuals (sensitivity ≥50%) and should have very high specificity (≥99%) [66].

Our results clearly show that the analytical performances of the study Ag-RDST were better in the event of a high viral load, i.e., in the case of significant viral excretion, especially in symptomatic individuals. These observations confirm the differential interest of Ag-RDT for the detection of SARS-CoV-2 antigens depending on the level of viral load of the sample analyzed, underlined by several authors [67,68,69], and international [52] and national [66,70] recommendations. As stated for Ag-RDT [52,66,70], Ag-RDSTs may be used at best to detect SARS-CoV-2 infected symptomatic individuals suffering from COVID-19-like symptoms with high viral loads, thus highly contagious individuals.

Previous studies to date have looked at the performance of unobserved as well as supervised self-collected specimens for molecular detection of SARS-CoV-2 [45,71,72,73,74,75,76,77]. Thus, Tu and colleagues compared SARS-CoV-2 detection using a variety of swab types self-collected (under health care professional observation) by 530 symptomatic child and adult outpatients (or their parents or guardians), with nasopharyngeal swabs collected by health care professionals as the comparator gold standard [72]. Of the nasopharyngeal swabs collected by the health care professional, 51 (9.6%) were positive. Mid-turbinate self-collected specimens showed high sensitivity of 96.2% (87.0% to 100%) [72]. McCulloch and colleagues have shown that unsupervised home mid-nasal swab collection was comparable to clinician-collected nasopharyngeal swab collection for molecular detection of SARS-CoV-2 in symptomatic patients, particularly those with higher viral loads [71]. The high analytical performance of self-collected samples, including NMT samples, for the molecular detection of common respiratory viruses was similarly reported [44,45,46,47,48,78]. A meta-analysis of nine studies comparing self-sampling and health care worker sampling for influenza testing reported a pooled sensitivity of 87% and specificity of 99% for self-collection [79].

Finally, the performances of supervised self-collected specimens for detection of SARS-CoV-2 using a WHO-listed SARS-CoV-2 Ag-RDT was evaluated in adult volunteers in Charité hospital, Universitätsmedizin Berlin, Germany [12,13,14,15]. Thus, nasal sampling (including self-sampling) assessed against nasopharyngeal sampling led to comparable performance using SARS-CoV-2 Ag-RDT [12,13,15]. Klein and colleagues extended these studies by evaluating the clinical performances of a supervised, self-collected NMT swab and a professional-collected naso-pharyngeal swab, using Panbio™ Ag-RDT (distributed by Abbott) by reference to molecular testing [14]. Participants were able to reliably carry out the NMT-self-sampling. A SARS-CoV-2 infection was diagnosed by rtRT-PCR in 45 of 290 participants (15.5%). Comparing the NMT and naso-pharyngeal sampling, the positive percent agreement of the Ag-RDT was 88.1% (95% CI 75.0–94.8%). The sensitivity of Panbio™ Ag-RDT was between 84.4% and 88.9%. Specificity was >99.0% for NMT and naso-pharyngeal sampling. The sensitivity of the Panbio™ Ag-RDT in participants with high viral load (>7 log10 SARS-CoV-2 RNA copies/mL) was 96.3% (95% CI 81.7–99.8%) for both NMT and naso-pharyngeal sampling. Supervised NMT self-sampling yielded comparable results to NP sampling. Overall, our findings and the Klein’s study demonstrate that NMT self-sampling leads to results comparable to nasopharyngeal sampling when using a convenient Ag-RDT. The possible reduction in SARS-CoV-2 viral load present in the nasal region compared to the nasopharyngeal region may be counterbalanced by the ease-of-sampling of nasal self-collection, as hypothesized [13]. In the same setting, Nikolai and colleagues further showed that professional anterior nasal and NMT sampling were of equivalent accuracy for a SARS-CoV-2 Ag-RDT in ambulatory symptomatic adults, but did not evaluate anterior nasal self-sampling for Ag-RDST [13]. Interestingly, the Centers for Disease Control and Prevention (Atlanta, GA, USA) have added recently both self-sampling for analysis in a reference laboratory and home, self NMT-sampling as an acceptable alternative to professional nasopharyngeal-sampling in their guidance for SARS-CoV-2 testing [11]. Direct COVID-19 self-tests and home tests that have received Emergency Use Authorization (EUA) status from the U.S. Food and Drug Administration (FDA) and are on the market, and are even available in common pharmacies in the USA. Oral fluid-based self-tests have also been authorized by the FDA under an EUA, although further clinical studies are warranted on the sensitivity of saliva as sample material for COVID-19 self-testing.

Other technologies under development could be used as a self-test. For example, loop-mediated isothermal amplification (LAMP) is a DNA amplification method that allows rapid and sensitive detection of a specific gene. LAMP merged with reverse transcription (RT-LAMP) has been successfully used for the detection of several respiratory RNA viruses, including SARS-CoV-2 [80]. Recently, a low cost, ease of use, paper-based device has been developed for extraction-free detection of SARS-CoV-2 in whole saliva using RT-LAMP with a colorimetric response visible to the human eye, that could be used as a self-test or in home diagnostics because of its simplicity [81].

### 4.3. Strengths and Limitations

Our study is original by highlighting both the usability assessed by several complementary sub-studies and the clinical performance of a novel NMT self-sampling-based Ag-RDST to diagnose SARS-CoV-2 infection in a real-setting in Paris during the third COVID-19 epidemic in France. Using written and illustrated instructions, participants were able to easily carry out NMT-self-sampling. Furthermore, procedural sampling-deviations might be reduced by video instructions. NMT collection was performed just after health care professional collection, allowing accurate comparisons. All samples for routine molecular diagnosis of SARS-CoV-2 infection were tested via the same rtRT-PCR assay. However, the study has some limitations. First, there was only one inclusion site, that could be the basis for allocation bias during randomization. Second, the presence of an observer may lead to a bias in our observations concerning the participants’ ability to perform the tests and to interpret the results. Third, participants who performed self-sampling were predominantly young and educated adults. The study was however limited by the lack of adolescents or children included. Fourth, the low sample size of included participants could reduce the study’s power, especially to evaluate the analytical performance of the Ag-RDST, as previously shown for test at a Tier 1 performance level (e.g., sensitivity ≥90% and specificity ≥95%) [82]. Finally, the study Ag-RDST characteristics might be different depending on whether an individual had previously tested positive, or has knowledge on self-testing for other conditions.

## 5. Conclusions

Ag-RDST may be used to detect SARS-CoV-2 infected symptomatic individuals suffering from COVID-19-like symptoms with high viral loads and has the potential to determine highly contagious individuals [52,66,70]. Large screening of populations with active SARS-CoV-2 infection is a possible way to break chains of transmission to limit the current pandemic and deconfine societies. While repeated population screening of asymptomatic individuals can be used to limit the spread of SARS-CoV-2, the speed of reporting is much more important than sensitivity, because the results concerning the screening of infected and asymptomatic individuals must always be known quickly [18]. Thus, despite a lower sensitivity to detect infection, Ag-RDST for SARS-CoV-2 antigen detection can be an important tool for screening because of their quick turnaround time and lower costs [67,68]. The rapid realization of Ag-RDST can help limit the transmission of SARS-CoV-2 by more quickly identifying infectious people to isolate, especially when used in the setting of serial testing strategies.

The need for health services has grown worldwide during the COVID-19 pandemic. Innovative ways to address this crisis are required. Self-collection of nasal swabs for SARS-CoV-2 antigenic testing offers an acceptable and reliable alternative to health worker collected samples. Thus, self-swab collection associated with Ag-RDST presents several advantages, which are even more important at a time of global health crisis, including accessibility outside of the health care system, minimizing personal protective equipment use, limiting exposure of patients and staff to infection, and providing a more comfortable patient experience. Self-collection for SARS-CoV-2-infected or COVID-19 patients is well-accepted, safe, and scalable in the pandemic setting, permitting widespread testing of both asymptomatic and symptomatic participants early in the illness and the potential for prompt self-quarantine and contract tracing [16]. The high sensitivities of Ag-RDST, especially in symptomatic patients, alleviate concerns of increased false negatives in this context. As societies reopen, expansion of testing is become critical for preventing a global resurgence in COVID-19. Nasal self-sampling with Ag-RDST has the potential to play a pivotal role in increasing testing access across the broader population.

## Figures and Tables

**Table 1:** Demographic characteristics and medical history of the 106 study participants.

Variable	Items	Number (%)
Sex		
	Male	38 (35.8)
	Female	68 (64.2)
Age (years)		
	18–20	4 (3.7)
	21–59	96 (90.6)
	≥60	6 (5.7)
Educational level		
	No diploma	17 (16.1)
	College level	40 (37.7)
	High school level	22 (20.7)
	Post-graduate level	27 (25.5)
Previous use of other		
	self-tests (not for COVID-19)	
	Yes	13 (12.3)
	No	93 (87.7)
Asymptomatic for COVID-19		
	Yes	76 (71.7)
	Contact case	10 (9.4)
Symptomatic for COVID-19 ^#^		
	Yes	30 (28.3)
Day from the onset		
	0–1	7 (6.6)
	2–4	20 (18.8)
	5–7	3 (2.9)
	>7	0 (0.0)

^#^ Participants who reported having at least one of the following major symptoms associated or not with minor symptoms were considered to have a COVID-19 symptom: fever, fatigue, dry cough, anosmia, and dyspnea. Minor symptoms were: pain, nasal congestion, runny nose, sore throat or diarrhea.

**Table 2:** Analytical results of the evaluation questionnaire concerning the ability of the 106 study participants to understand the instruction for use of the BIOSYNEX Antigen Self-Test COVID-19 Ag+ (Biosynex Swiss SA).

Comprehension of Labeling Checklist *	Participants’ Responses		
	True [Number (%)]	False [Number (%)]	Do Not Know [Number (%)]
Q1: “The same swab is used to take both nostrils one after the other”	106 (100)	0 (0.0)	0 (0.0)
Q2: “The nasal swab is inserted into one nostril until silk resistance is reached (approximately 3 cm)”	104 (98.2)	0 (0.0)	2 (1.8)
Q3: “The nasal swab must touch the walls of the nostril”	100 (94.3)	1 (0.9)	5 (4.7)
Q4: “The swab has to be turned 6 times in the tube filled with diluent (during at least 10 s)”	106 (100)	0 (0.0)	0 (0.0)
Q5: “The swab should be properly wrung out in the tube to let a maximum of nasal sample”	100 (94.3)	1 (0.9)	5 (4.7)
Q6: “A nipple must be placed on the tube before depositing the 4 drops of solution in the well “S””	104 (98.2)	1 (0.9)	1 (0.9)
Q7: “Lack of band by test results is interpreted as a negative test”	1 (0.9)	102 (96.2)	3 (2.8)
Q8: “Lack of control band by test results should be interpreted as an invalid test”	101 (95.3)	2 (1.8)	3 (2.8)
Q9: “Having symptoms of fever, cough and aches, the negativity of BIOSYNEX Antigen Self-test COVID-19 Ag+ confirm the lack of COVID-19 infection”	2 (1.8)	103 (97.2)	1 (0.9)
Q10: “If the test is positive, it means that I have to see the doctor and additional tests will be required to confirm the diagnosis”	106 (100)	0 (0.0)	0 (0.0)
Q11: “The BIOSYNEX Antigen Self-test COVID-19 Ag+ detects the presence of the virus”	104 (98.2)	0 (0.0)	2 (1.8)
Labeling index for understanding (% [95% CI]) ^£^	96.8 [91.5–98.8]		
Correct understanding of the instruction for use (n; % [95% CI]) ^#^	100; 94.3 [88.3–97.3]		

* Overall, 76 (71.7%) participants preferred to use the paper-based instruction whereas 30 (28.3%) participants used the video-based instruction; ^£^ The labeling index for understanding was defined as the mean of the correct answers for each question; # The participants who correctly answered all 11 questions were considered to have correctly understood the instructions for use. CI: Confidence interval; COVID-19: Coronavirus disease 2019; Q: Question.

**Table 3:** Analytical results of the manipulation observation concerning the ability of Table, 106 study participants to correctly use each step of the BIOSYNEX Antigen Self-Test COVID-19 Ag+ (Biosynex Swiss SA) autonomously or with verbal help.

Usability Checklist ^µ^	Successful Manipulation		Need for Verbal Help
	Yes [Number (%)]	No [Number (%)]	Yes [Number (%)]
Did the participant open the box?	106 (100)	-	-
2. Did the participant read the instructions for use (written part and drawn part)?	106 (100)	-	-
3. Did the participant wash his hands?	104 (98.2)	2 (1.8)	1 (0.9)
4. Did the participant easily identify the different components of the kit?	106 (100)	-	-
5. Did the participant properly remove the test cassette from the aluminum pouch?	105 (99.1)	1 (0.9)	-
6. Did the participant open the lid and place the tube on the rack correctly?	104 (98.2)	2 (1.8)	2 (1.8)
7. Did the participant introduce his nasal swab correctly in both nostrils?	106 (100)	-	8 (7.5)
8. Did the participant turn the swab 6 times in the tube?	101 (95.3)	5 (4.7)	3 (2.8)
9. Did the participant wait for 1 min after turning the swab?	104 (98.2)	2 (1.8)	1 (0.9)
10. Did the participant properly wring out the tightness of the swab?	104 (98.2)	2 (1.8)	6 (5.7)
11. Did the participant correctly add the nipple to the tube?	106 (100)	-	-
12. Did the participant correctly deposit 4 drops into the “S” well of the test cassette?	106 (100)	-	-
13. Did the participant observe the correct time after deposition of the sample drops into the “S” well of the cassette?	106 (100)	-	-
14. Did the participant obtain an interpretable result at the end of the process? ^#^	106 (100)	-	3 (2.8)
Usability index (% [95% CI]) ^£^	99.1 [94.9–99.8]		
Correct use without difficulties, errors, or helps (n; % [95% CI])	92; 86.8 [79.1–92.0]		
Correct use with help (n; % [95% CI])	14; 13.2 [8.0–20.9]		
Average time of manipulation (minutes [SD])	8.1 [1.3]		

^µ^ A total of 30 (28.3%) participants used the video-based instruction for use; among them the usability index was estimated to 100% without any difficulties, errors, or help; ^#^ The result was considered interpretable when a control strip was readable after the migration time recommended by the manufacturer; in the present series, 21 (27.3%) participants had a positive self-test result; ^£^ The usability index was defined as the mean of the correct answers for each question. CI: Confidence interval; SD: Standard deviation.

**Table 4:** Items and results of the satisfaction questionnaire and concerning the instruction notice (sub-study 1), the performing of the BIOSYNEX Antigen Self-Test COVID-19 Ag+ (Biosynex Swiss SA) (sub-study 2), and the interpretation of test results.

Satisfaction Questionnaire	Post-Test Satisfaction [Number (%)]
How did you find the understandability of instructions for use of self-test?	
Very easy	61 (57.5)
Rather easy	39 (36.8)
Rather difficult	6 (5.7)
Very difficult	0 (0)
How did you find the overall implementation of the self-test kit?	
Very easy	53 (50.0)
Rather easy	43 (40.6)
Rather difficult	9 (8.5)
Very difficult	1 (0.9)
How did you find the sample collection with nasal swabbing in both nostrils?	
Very easy	37 (34.9)
Rather easy	59 (55.7)
Rather difficult	8 (7.7)
Very difficult	2 (1.8)
How did you find the use of the component of the self-test kit and the sample deposition into the “S” well of the test cassette?	
Very easy	53 (50.0)
Rather easy	44 (41.4)
Rather difficult	8 (7.7)
Very difficult	1 (0.9)
How did you find the reading of strips after migration?	
Very easy	89 (83.9)
Rather easy	12 (11.4)
Rather difficult	5 (4.7)
Very difficult	0 (0)
How did you find the interpretation of self-test results?	
Very easy	95 (89.6)
Rather easy	11 (10.4)
Rather difficult	0 (0)
Very difficult	0 (0)
How did you find the video on YouTube to help with performing the self-test? (*n* = 30)	
Very easy	24 (80.0)
Rather easy	6 (20.0)
Rather difficult	0 (0)
Very difficult	0 (0)
How did you find the overall handling of the self-test component?	
Very easy	80 (75.5)
Rather easy	17 (16.0)
Rather difficult	9 (8.5)
Very difficult	0 (0)
How did you find your ability to surmount the difficulties encountered?	
Very easy	100 (94.4)
Rather easy	6 (5.6)
Rather difficult	0 (0)
Very difficult	0 (0)
You have just performed the nasal COVID-19 self-test, do you repeat it if necessary?	
Yes	83 (78.3)
No	23 (21.7)
You just took the COVID-19 nasal self-test; would you recommend it to someone else?	
Yes	99 (93.4)
No	7 (6.6)

**Table 5:** Analytical performance of the BIOSYNEX Antigen Self-Test COVID-19 Ag+ for the qualitative detection of the N protein of SARS-CoV-2 using 106 prospectively collected nasopharyngeal swab samples by reference rRT-PCR#.

				BIOSYNEX COVID-19 Ag BSS ^§^								
		Mean Genes C_t_ *^,#^ (Median; Range)	N	TN *(n)*	FN *(n)*	TP *(n)*	FP *(n)*	Sensitivity (% [95% CI]) ^µ^	Specificity (% [95% CI])	Agreement ^a^	Concordance ^b^	Youden’s J Index ^c^
PCR	Symptomatic (n = 30)	23.1 (19.5–30.0)	16	0	1	15	0	93.8 (79.3–98.4)	100 (88.6–100)	96.7 (83.4–99.4)	0.93 (0.78–0.98)	93.8 (79.3–98.4)
		Negative	14	14	0	0	0					
	Asymptomatic (*n* = 76)	28.2 (25.0–33.0)	6	0	1	5	0	83.3 (73.4–90.0)	100 (95.2–100)	98.7 (92.0–99.6)	0.91 (0.82–0.96)	83.3 (73.4–90.0)
		Negative	70	70	0	0	0					
	Whole study population (*n* = 106)	26.2 (19.5–33.0)	22	0	2	20	0	90.9 (83.9–95.0)	100 (96.5–100)	98.1 (93.4–99.5)	0.94 (0.88–0.97)	90.9 (83.9–95.0)
		Negative	84	84	0	0	0					

^§^ Nasopharyngeal samples in one nostril were collected with a flocked swab for each volunteer participant by trained healthcare personnel (nurses or biologists), while the swab contained in the BIOSYNEX Antigen Self-Test COVID-19 Ag+ kit was further used for nasal secretions self-sampling by the participant himself. Molecular testing as well COVID-19 antigen detection were carried out on fresh samples; ^a^ Agreement = TP + TN/TP + FP + TN + FN, expressed in percentage; ^b^ The Cohen’s κ coefficient calculation was used to estimate the concordance [35] and interpreted according the Landis and Koch scale [36], as follows: < 0 as indicating no agreement, 0–0.20 as slight, 0.21–0.40 as fair, 0.41–0.60 as moderate, 0.61–0.80 as substantial, and 0.81–1 as almost perfect concordance; ^c^ The accuracy of the test BIOSYNEX COVID-19 Ag BSS to correctly diagnose SARS-CoV-2 infection was estimated by Youden’s J index (J = sensitivity + specificity − 1) [37]; ^µ^ 95% confidence intervals in brackets were calculated by using the Wilson score bounds; ^#^ The CE IVD-marked BIOSYNEX AmpliQuick^®^ SARS-CoV-2 (Biosynex, Strasbourg, France) constituted the reference multiplex rRT-PCR for SARS-CoV-2 RNA detection. This assay detects two target genes of SARS-CoV-2 (E and RdRP genes); * Mean genes Ct = mean of Ct for E gene and Ct for RdRP gene. Ct: Cycle threshold; FN: False negative; FP: False positive; NA: Not attributable; rRT-PCR: real-time reverse transcription-polymerase chain reaction; TP: True positive; TN: True negative.

## Supplementary Materials

The following are available online at www.mdpi.com/article/10.3390/diagnostics11122217/s1, Figure S1: Instruction for use of BIOSYNEX Antigen Self-Test COVID-19 Ag+.

## References

[1] Hardan L., Filtchev D., Kassem R., Bourgi R., Lukomska-Szymanska M., Tarhini H., Salloum-Yared F.D., Mancino D., Kharouf N., Haikel Y. (2021). COVID-19 and Alzheimer’s Disease: A Literature Review. Medicina.

[2] Grassly N.C., Pons-Salort M., Parker E.P.K., White P.J., Ferguson N.M., Imperial College COVID-19 Response Team (2020). Comparison of molecular testing strategies for COVID-19 control: A mathematical modelling study. Lancet Infect. Dis..

[3] Paltiel A.D., Zheng A., Walensky R.P. (2020). Assessment of SARS-CoV-2 Screening Strategies to Permit the Safe Reopening of College Campuses in the United States. JAMA Netw. Open.

[4] Smithgall M.C., Dowlatshahi M., Spitalnik S.L., Hod E.A., Rai A.J. (2020). Types of Assays for SARS-CoV-2 Testing: A Review. Lab. Med..

[5] Rai P., Kumar B.K., Deekshit V.K., Karunasagar I., Karunasagar I. (2021). Detection technologies and recent developments in the diagnosis of COVID-19 infection. Appl. Microbiol. Biotechnol..

[6] Dinnes J., Deeks J.J., Adriano A., Berhane S., Davenport C., Dittrich S., Emperador D., Takwoingi Y., Cunningham J., Beese S. (2020). Rapid, point-of-care antigen and molecular-based tests for diagnosis of SARS-CoV-2 infection. Cochrane Database Syst. Rev..

[7] Li D., Li J. (2020). Immunologic testing for SARS-CoV-2 infection from the antigen perspective. J. Clin. Microbiol..

[8] European Centre for Disease Prevention and Control Options for the Use of Rapid Antigen Tests for COVID-19 in the EU/EEA and the UK. 19 November 2020. https://www.ecdc.europa.eu/en/publications-data/options-use-rapid-antigen-tests-covid-19-eueea-and-uk.

[9] Toptan T., Eckermann L., Pfeiffer A.E., Hoehl S., Ciesek S., Drosten C., Corman V.M. (2020). Evaluation of a SARS-CoV-2 rapid antigen test: Potential to help reduce community spread?. J. Clin. Virol..

[10] Deitmer T., Dietz A., Chaberny I.F., Pietsch C. (2021). [The nasal and pharyngeal swab techniques during the COVID-19-pandemic-the ENT-perspective-SARS-CoV-2, Coronavirus, nasal swab, pharyngeal swab, complications]. Laryngo-Rhino-Otologie.

[11] Centers for Disease Control and Prevention (CDC) Interim Guidelines for Collecting, Handling, and Testing Clinical Specimens for COVID-19. Updated 26 February 2021. https://www.cdc.gov/coronavirus/2019-ncov/lab/guidelines-clinical-specimens.html.

[12] Lindner A.K., Nikolai O., Kausch F., Wintel M., Hommes F., Gertler M., Krüger L.J., Gaeddert M., Tobian F., Lainati F. (2021). Head-to-head comparison of SARS-CoV-2 antigen-detecting rapid test with self-collected nasal swab versus professional-collected nasopharyngeal swab. Eur. Respir J..

[13] Nikolai O., Rohardt C., Tobian F., Junge A., Corman V.M., Jones T.C., Gaeddert M., Lainati F., Sacks J.A., Seybold J. (2021). Anterior nasal versus nasal mid-turbinate sampling for a SARS-CoV-2 antigen-detecting rapid test: Does localisation or professional collection matter?. MedRxiv.

[14] Klein J.A.F., Krüger L.J., Tobian F., Gaeddert M., Lainati F., Schnitzler P., Lindner A.K., Nikolai O., Knorr B., Welker A. (2021). Head-to-head performance comparison of self-collected nasal versus professional-collected nasopharyngeal swab for a WHO-listed SARS-CoV-2 antigen-detecting rapid diagnostic test. Med. Microbiol. Immunol..

[15] Krüger L.J., Klein J.A.F., Tobian F., Gaeddert M., Lainati F., Klemm S., Schnitzler P., Bartenschlager R., Cerikan B., ACE-IT Study Group (2021). Evaluation of accuracy, exclusivity, limit-of-detection and ease-of-use of LumiraDx™: An antigen-detecting point-of-care device for SARS-CoV-2. Infection.

[16] Liao W.T., Hsu M.Y., Shen C.F., Hung K.F., Cheng C.M. (2020). Home Sample Self-Collection for COVID-19 Patients. Adv. Biosyst..

[17] Boum Y., Eyangoh S., Okomo M.C. (2021). Beyond COVID-19-will self-sampling and testing become the norm?. Lancet Infect. Dis..

[18] Larremore D.B., Wilder B., Lester E., Shehata S., Burke J.M., Hay J.A., Tambe M., Mina M.J., Parker R. (2020). Test sensitivity is secondary to frequency and turnaround time for COVID-19 screening. Sci. Adv..

[19] Mercer T.R., Salit M. (2021). Testing at scale during the COVID-19 pandemic. Nat. Rev. Genet..

[20] Peacock F.W., Dzieciatkowski T., Chirico F., Szarpak L. (2021). Self-testing with antigen tests as a method for reduction SARS-CoV-2. Am. J. Emerg. Med..

[21] Goggolidou P., Hodges-Mameletzis I., Purewal S., Karakoula A., Warr T. (2021). Self-Testing as an Invaluable Tool in Fighting the COVID-19 Pandemic. J. Prim. Care Community Health.

[22] Figueroa C., Johnson C., Ford N., Sands A., Dalal S., Meurant R., Prat I., Hatzold K., Urassa W., Baggaley R. (2018). Reliability of HIV rapid diagnostic tests for self-testing compared with testing by health-care workers: A systematic review and meta-analysis. Lancet HIV.

[23] Rivera A.S., Hernandez R., Mag-Usara R., Sy K.N., Ulitin A.R., O’Dwyer L.C., McHugh M.C., Jordan N., Hirschhorn L.R. (2021). Implementation outcomes of HIV self-testing in low- and middle- income countries: A scoping review. PLoS ONE.

[24] FDA News Release. Coronavirus (COVID-19) Update: FDA Issues New Authorization for the BinaxNOW COVID-19 Ag Card Home Test. 16 December 2020. https://www.fda.gov/news-events/press-announcements/coronavirus-covid-19-update-fda-issues-new-authorization-binaxnow-covid-19-ag-card-home-test.

[25] Osmanodja B., Budde K., Zickler D., Naik M.G., Hofmann J., Gertler M., Hülso C., Rössig H., Horn P., Seybold J. (2021). Accuracy of a Novel SARS-CoV-2 Antigen-Detecting Rapid Diagnostic Test from Standardized Self-Collected Anterior Nasal Swabs. J. Clin. Med..

[26] Stohr J.J.J.M., Zwart V.F., Goderski G., Meijer A., Nagel-Imming C.R.S., Kluytmans-van den Bergh M.F.Q., Pas S.D., van den Oetelaar F., Hellwich M., Gan K.H. (2021). Self-testing for the detection of SARS-CoV-2 infection with rapid antigen tests for people with suspected COVID-19 in the community. Clin. Microbiol. Infect..

[27] World Health Organization (WHO) WHO prequalification: Sample Product Dossier for an IVD Intended for HIV Self-Testing. SIMU™ Self-Test for HIV 12O Working Document, December 2015..

[28] Prazuck T., Karon S., Gubavu C., Andre J., Legall J.M., Bouvet E., Kreplak G., Teglas J.P., Pialoux G. (2016). A finger-stick whole-blood HIV self-test as an HIV screening tool adapted to the general public. PLoS ONE.

[29] Tonen-Wolyec S., Batina-Agasa S., Muwonga J., Fwamba N’kulu F., Mboumba Bouassa R.S., Belec L. (2018). Evaluation of the practicability and virological performance of finger-stick whole-blood HIV self-testing in French-speaking sub-Saharan Africa. PLoS ONE.

[30] Tonen-Wolyec S., Dupont R., Batina-Agasa S., Hayette M.P., Bélec L. (2020). Capillary whole-blood IgG-IgM COVID-19 self-test as a serological screening tool for SARS-CoV-2 infection adapted to the general public. PLoS ONE.

[31] Journal Officiel de la République Française. Arrêté du 16 Octobre 2020 Modifiant l’arrêté du 10 Juillet 2020 Prescrivant les Mesures Générales Nécessaires Pour Faire Face à l’épidémie de Covid-19 Dans les Territoires Sortis de l’état d’urgence Sanitaire et Dans Ceux où il a été Prorogé—Légifrance JORF n°0253 du 17 Octobre 2020. https://www.legifrance.gouv.fr/eli/arrete/2020/10/16/SSAZ2027698A/jo/article_snum.

[32] Journal Officiel de la République Française. Arrêté du 12 Décembre 2020 Portant Modification des Conditions de Remboursement de l’acte de Détection du Génome du SARS-CoV-2 Par Amplification Génique. https://www.legifrance.gouv.fr/download/file/MG2FA_IlBEW4Y-eXqzLe7od0-jy1YneS77Jhrh9N7pM=/JOE_TEXTE.

[33] Haute Autorité de Santé, Saint-Denis, France, 6 mars 2020. Avis n°2020.0020/AC/SEAP du 6 Mars 2020 du Collège de la HAS relatif à l’inscription Sur la LAP Mentionnée à l’article L. 162-1-7 du CSS, de la Détection du Génome du Coronavirus SARS-CoV-2 par Technique de Transcription Inverse Suivie d’une Amplification. https://www.has-sante.fr/jcms/p_3161218/fr/avis-n2020-0020/ac/seap-du-6-mars-2020-du-college-de-la-has-relatif-a-l-inscription-sur-la-lap-mentionnee-a-l-article-l-162-1-7-du-css-de-la-detection-du-genome-du-coronavirus-sars-cov-2-par-technique-de-transcription-inverse-suivie-d-une-amplification.

[34] Newcombe R.G. (1998). Two-sided confidence intervals for the single proportion: Comparison of 362 seven methods. Stat. Med..

[35] Cohen J. (1960). A coefficient of agreement for nominal scales. Educ. Psychol. Meas..

[36] Landlis J.R., Koch G.G. (1977). The measurement of observer agreement for categorical data. Biometrics.

[37] Youden W.J. (1950). Index for rating diagnostic tests. Cancer.

[38] Journal Officiel de la République Française. Ordonnance n° 2010-49 du 13 Janvier 2010 Relative à la Biologie Médicale. https://www.legifrance.gouv.fr/jorf/id/JORFTEXT000021683301/.

[39] Haute Autorité de Santé, Saint-Denis, France, 23 avril 2021. Avis n° 2021.0029/AC/SEAP du 23 Avril 2021 du Collège de la HAS Relatif à la Détection Antigénique Rapide du Virus SARS-CoV-2 sur Prélèvement Nasal (TDR, TROD et Autotest). https://www.has-sante.fr/jcms/p_3263368/fr/avis-n-2021-0029/ac/seap-du-23-avril-2021-du-college-de-la-has-relatif-a-la-detection-antigenique-rapide-du-virus-sars-cov-2-sur-prelevement-nasal-tdr-trod-et-autotest.

[40] Tonen-Wolyec S., Mboup S., Grésenguet G., Bouassa R.B., Bélec L. (2018). Insufficient education is a challenge for HIV self-testing. Lancet HIV.

[41] World Health Organization Guidelines on HIV Self-Testing and Partner Notification: Supplement to Consolidated Guidelines on HIV Testing Services. December 2016..

[42] Ortblad K.F., Musoke D.K., Ngabirano T., Nakitende A., Haberer J.E., McConnell M., Salomon J.A., Bärnighausen T., Oldenburg C.E. (2018). Female sex workers often incorrectly interpret HIV self-test results in Uganda. J. Acquir. Immune Defic. Syndr..

[43] Tahlil K.M., Ong J.J., Rosenberg N.E., Tang W., Conserve D.F., Nkengasong S., Muessig K.E., Iwelunmor J., Ezechi O., Gbaja-biamila T. (2020). Verification of HIV Self-Testing Use and Results: A Global Systematic Review. AIDS Patient Care STDS.

[44] Larios O.E., Coleman B.L., Drews S.J., Mazzulli T., Borgundvaag B., Green K., McGeer A.J., STOP-Flu Study Group (2011). Self-collected mid-turbinate swabs for the detection of respiratory viruses in adults with acute respiratory illnesses. PLoS ONE.

[45] Cockerill F.R., Wohlgemuth J.G., Radcliff J., Sabol C.E., Kapoor H., Dlott J.S., Marlowe E.M., Clarke N.J. (2021). Evolution of Specimen Self-Collection in the COVID-19 Era: Implications for Population Health Management of Infectious Disease. Popul. Health Manag..

[46] Akmatov M.K., Gatzemeier A., Schughart K., Pessler F. (2012). Equivalence of self- and staff-collected nasal swabs for the detection of viral respiratory pathogens. PLoS ONE.

[47] Dhiman N., Miller R.M., Finley J.L. (2012). Effectiveness of patient-collected swabs for influenza testing. Mayo Clin. Proc..

[48] Goyal S., Prasert K., Praphasiri P. (2017). The acceptability and validity of self-collected nasal swabs for detection of influenza virus infection among older adults in Thailand. Influenza Other Respir. Viruses.

[49] Jackson M.L., Nguyen M., Kirlin B., Madziwa L. (2015). Self-collected nasal swabs for respiratory virus surveillance. Open Forum Infect. Dis..

[50] Prazuck T., Phan Van J., Sinturel F., Levray F., Elie A., Camera D., Pialoux G. (2021). Evaluation of the practicability of a finger-stick whole-blood SARS-Cov-2 self-test adapted for the general population. PLoS ONE.

[51] Zou L., Ruan F., Huang M., Liang L., Huang H., Hong Z., Yu J., Kang M., Song Y., Xia J. (2020). SARS-CoV-2 Viral Load in Upper Respiratory Specimens of Infected Patients. N. Engl. J. Med..

[52] World Health Organization Interim Guidance. Antigen-Detection in the Diagnosis of SARS-CoV-2 Infection Using Rapid Immune-Assays. 11 September 2020..

[53] Cerutti F., Burdino E., Milia M.G., Allice T., Gregori G., Bruzzone B., Ghisetti V. (2020). Urgent need of rapid tests for SARS CoV-2 antigen detection: Evaluation of the SD-Biosensor antigen test for SARS-CoV-2. J. Clin. Virol..

[54] Chaimayo C., Kaewnaphan B., Tanlieng N., Athipanyasilp N., Sirijatuphat R., Chayakulkeeree M., Angkasekwinai N., Sutthent R., Puangpunngam N., Tharmviboonsri T. (2020). Rapid SARS-CoV-2 antigen detection assay in comparison with real-time RT-PCR assay for laboratory diagnosis of COVID-19 in Thailand. Virol. J..

[55] Diao B., Wen K., Zhang J., Chen J., Han C., Chen Y., Wang S., Deng G., Zhou H., Wu Y. (2020). Accuracy of a nucleocapsid protein antigen rapid test in the diagnosis of SARS-CoV-2 infection. Clin. Microbiol. Infect..

[56] Linares M., Pérez-Tanoira R., Carrero A., Romanyk J., Pérez-García F., Gómez-Herruz P., Arroyo T., Cuadros J. (2020). Panbio antigen rapid test is reliable to diagnose SARS-CoV-2 infection in the first 7 days after the onset of symptoms. J. Clin. Virol..

[57] Weitzel T., Legarraga P., Iruretagoyena M., Pizarro G., Vollrath V., Araos R., Munita J.M., Porte L. (2020). Comparative evaluation of four rapid SARS-CoV-2 antigen detection tests using universal transport medium. Travel Med. Infect. Dis..

[58] Courtellemont L., Guinard J., Guillaume C., Giaché S., Rzepecki V., Seve A., Gubavu C., Baud K., Le Helloco C., Cassuto G.N. (2021). High performance of a novel antigen detection test on nasopharyngeal specimens for diagnosing SARS-CoV-2 infection. J. Med. Virol..

[59] Mboumba Bouassa R.S., Veyer D., Péré H., Bélec L. (2021). Analytical performances of the point-of-care SIENNA™ COVID-19 Antigen Rapid Test for the detection of SARS-CoV-2 nucleocapsid protein in nasopharyngeal swabs: A prospective evaluation during the COVID-19 s wave in France. Int. J. Infect. Dis..

[60] Favresse J., Gillot C., Oliveira M., Cadrobbi J., Elsen M., Eucher C., Laffineur K., Rosseels C., Van Eeckhoudt S., Nicolas J.B. (2021). Head-to-Head Comparison of Rapid and Automated Antigen Detection Tests for the Diagnosis of SARS-CoV-2 Infection. J. Clin. Med..

[61] Schildgen V., Demuth S., Lüsebrink J., Schildgen O. (2021). Limits and Opportunities of SARS-CoV-2 Antigen Rapid Tests: An Experienced-Based Perspective. Pathogens.

[62] Albert E., Torres I., Bueno F., Huntley D., Molla E., Fernández-Fuentes M.Á., Martínez M., Poujois S., Forqué L., Valdivia A. (2021). Field evaluation of a rapid antigen test (Panbio COVID-19 Ag Rapid Test Device) for COVID-19 diagnosis in primary healthcare centres. Clin. Microbiol. Infect..

[63] Scohy A., Anantharajah A., Bodéus M., Kabamba-Mukadi B., Verroken A., Rodriguez-Villalobos H. (2020). Low performance of rapid antigen detection test as frontline testing for COVID-19 diagnosis. J. Clin. Virol..

[64] Yamayoshi S., Sakai-Tagawa Y., Koga M., Akasaka O., Nakachi I., Koh H., Maeda K., Adachi E., Saito M., Nagai H. (2020). Comparison of Rapid Antigen Tests for COVID-19. Viruses.

[65] Osterman A., Baldauf H.M., Eletreby M., Wettengel J.M., Afridi S.Q., Fuchs T., Holzmann E., Maier A., Döring J., Grzimek-Koschewa N. (2021). Evaluation of two rapid antigen tests to detect SARS-CoV-2 in a hospital setting. Med. Microbiol. Immunol..

[66] Haute Autorité de Santé, Saint-Denis, France, 8 octobre 2020. Revue Rapide sur les Tests de Détection Antigénique du Virus SARS-CoV-2. File:///C:/Users/prbel/Downloads/synthese_tests_antigeniques_vd%20(1).pdf.

[67] Guglielmi G. (2020). Fast coronavirus tests: What they can and can’t do. Nature.

[68] Mattiuzzi C., Henry B., Lippi G. (2020). Making sense of rapid antigen testing in SARS-CoV-2 diagnostics. Diagnosis.

[69] Mina M.J., Parker R., Larremore D.B. (2020). Rethinking Covid-19 Test Sensitivity—A Strategy for Containment. N. Engl. J. Med..

[70] Food and Drug Administration (2020). In Vitro diagnostics EUAs. Silver Spring, MD: US Department of Health and Human Services, Food and Drug Administration. https://www.fda.gov/medical-devices/coronavirusdisease-2019-covid-19-emergency-use-authorizations-medical-devices/vitro-diagnostics-euas.

[71] McCulloch D.J., Kim A.E., Wilcox N.C., Logue J.K., Greninger A.L., Englund J.A., Chu H.Y. (2020). Comparison of Unsupervised Home Self-collected Midnasal Swabs With Clinician-Collected Nasopharyngeal Swabs for Detection of SARS-CoV-2 Infection. JAMA Netw. Open.

[72] Tu Y.P., Jennings R., Hart B., Cangelosi G.A., Wood R.C., Wehber K., Verma P., Vojta D., Berke E.M. (2020). Swabs Collected by Patients or Health Care Workers for SARS-CoV-2 Testing. N. Engl. J. Med..

[73] Wehrhahn M.C., Robson J., Brown S., Bursle E., Byrne S., New D., Chong S., Newcombe J.P., Siversten T., Hadlow N. (2020). Self-collection: An appropriate alternative during the SARS-CoV-2 pandemic. J. Clin. Virol..

[74] Kagan R.M., Rogers A.A., Borillo G.A., Clarke N.J., Marlowe E.M. (2021). Performance of Unobserved Self-Collected Nasal Swabs for Detection of SARS-CoV-2 by RT-PCR Utilizing a Remote Specimen Collection Strategy. Open Forum Infect. Dis..

[75] Kriegova E., Fillerova R., Raska M., Manakova J., Dihel M., Janca O., Sauer P., Klimkova M., Strakova P., Kvapil P. (2021). Excellent option for mass testing during the SARS-CoV-2 pandemic: Painless self-collection and direct RT-qPCR. Virol. J..

[76] Torretta S., Zuccotti G., Cristofaro V., Ettori J., Solimeno L., Battilocchi L., D’Onghia A., Bonsembiante A., Pignataro L., Marchisio P. (2021). Diagnosis of SARS-CoV-2 by RT-PCR Using Different Sample Sources: Review of the Literature. Ear Nose Throat J..

[77] Tsang N.N.Y., So H.C., Ng K.Y., Cowling B.J., Leung G.M., Ip D.K.M. (2021). Diagnostic performance of different sampling approaches for SARS-CoV-2 RT-PCR testing: A systematic review and meta-analysis. Lancet Infect. Dis..

[78] Esposito S., Molteni C.G., Daleno C., Valzano A., Tagliabue C., Galeone C., Milani G., Fossali E., Marchisio P., Principi N. (2010). Collection by trained pediatricians or parents of mid-turbinate nasal flocked swabs for the detection of influenza viruses in childhood. Virol. J..

[79] Seaman C.P., Tran L.T.T., Cowling B.J., Sullivan S.G. (2019). Self-collected compared with professional-collected swabbing in the diagnosis of influenza in symptomatic individuals: A meta-analysis and assessment of validity. J. Clin. Virol..

[80] Davidson J.L., Wang J., Maruthamuthu M.K., Dextre A., Pascual-Garrigos A., Mohan S., Putikam S.V.S., Osman F.O.I., McChesney D., Seville J. (2021). A paper-based colorimetric molecular test for SARS-CoV-2 in saliva. Biosens. Bioelectron. X.

[81] Tompson D., Lei Y. (2020). Recent progress in RT-LAMP enabled COVID-19 detection. Sens. Actuators Rep..

[82] Kost G.J. (2021). The Impact of Increasing Disease Prevalence, False Omissions, and Diagnostic Uncertainty on Coronavirus Disease 2019 (COVID-19) Test Performance. Arch. Pathol. Lab. Med..

