# Supplementary material for: Evaluation of the Practicability of Biosynex Antigen Self-Test COVID-19 AG+ for the Detection of SARS-CoV-2 Nucleocapsid Protein from Self-Collected Nasal Mid-Turbinate Secretions in the General Public in France
Source: Diagnostics (Basel). 2021 Nov 27;11(12):2217. doi: 10.3390/diagnostics11122217 (PMC8700066; doi:10.3390/diagnostics11122217)
Supplement: Supplementary file 1 [file diagnostics-11-02217-s001.zip › diagnostics-1471335-supplementary.pdf]

BIOSYNEX

Autotest antigénique COVID-19 Ag+

FR

Test destiné à l'autodiagnostic.

INTRODUCTION

La COVID-19 est une maladie respiratoire aigüe causée par l'infection par le SARS-CoV-2. Le SARS-CoV-2 appartient à la même famille que les virus responsables de Syndromes Respiratoires Aigus Sévères (SRAS).
Les symptômes les plus courants de la COVID-19 sont la fièvre, la fatigue et la toux sèche. Certains patients peuvent avoir des douleurs, une congestion nasale, un écoulement nasal, un mal de gorge, la diarrhée, une perte du goût et de l'odorat. Ces symptômes sont généralement légers et commencent progressivement. Certaines personnes sont infectées mais ne développent aucun symptôme. La plupart des personnes (environ 80 %) se rétablissent de la maladie sans avoir besoin d'une prise en charge hospitalière. La maladie peut se transmettre d'individu en individu par de petites gouttelettes émises par le nez ou la bouche lorsqu'une personne infectée par le SARS-Cov-2, tousse ou expire. La transmission par contact des mains contaminées avec le visage est fréquente. Les mains peuvent en effet être contaminées après contact avec des surfaces porteuses du virus. La période estimée d'incubation du SARS-CoV-2 varie de 1 à 14 jours.

USAGE PRÉVU

Biosynex autotest antigénique COVID-19 Ag+ est un test rapide immunochromatographique in vitro qui permet la détection qualitative des antigènes de la protéine de la nucléocapside (N) du SARS-CoV-2 dans les échantillons de prélèvements nasaux. Il est conçu pour aider à établir le diagnostic rapide des infections par le SARS-CoV-2. Ce test est un test rapide immunochromatographique qualitatif qui utilise des anticorps monoclonaux hautement sensibles afin de détecter la protéine de la nucléocapside (N) du SARS-CoV-2.
Cet autotest permet de savoir si vous êtes actuellement infectés par le virus responsable de la COVID-19. Ce test est à utiliser uniquement chez les adultes ou sous la supervision d'un adulte si il est réalisé sur un enfant.
Un résultat négatif avec Biosynex autotest antigénique COVID-19 chez une personne asymptomatique n'exclut pas une infection à la COVID-19. En présence de symptômes évocateurs d'une COVID-19, un résultat négatif doit être contrôlé par un test en laboratoire.

COMPOSITION DU KIT

Pochette aluminium
(contient la cassette)

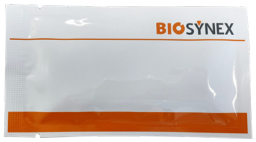

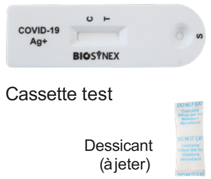

Cassette test

Dessicant
(à jeter)

Tube de diluant
pré-rempli

Tétine
compte-goutte

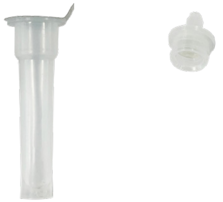

Dans un sachet transparent

Ecouvillon
CE0197 ou CE0123

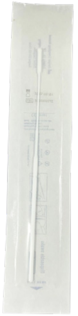

La réf. 859271 contient un sachet permettant de faire un autotest.
La réf. 859272 contient cinq sachets, chaque sachet permet de faire un autotest.

CONSEILS POUR LE PRÉLÈVEMENT

- Se laver les mains avant chaque test afin de diminuer le risque de contamination manuportée.
- Pour obtenir des résultats précis, ne pas utiliser des échantillons trop visqueux ou qui contiennent visiblement du sang. Il est possible de se moucher afin d'enlever l'excédent de mucus avant de réaliser le test.

STOCKAGE

- Ce test ne doit pas être congelé et doit être conservé dans un endroit sec entre 2°C et 30°C.
- La cassette de test doit rester dans le sachet scellé jusqu'à son utilisation.
- Ne pas utiliser l'autotest au-delà de la date de péremption figurant sur le sachet aluminium et sur l'extérieur de la boite en carton.

MISES EN GARDE DE MANIPULATION

- Tenir l'autotest et ses composants hors de portée des enfants ; le diluant peut présenter un danger en cas d'ingestion.
- La cassette de test usagée ainsi que tous les composants du test peuvent être jetés avec les déchets ménagers, dans un sac bien fermé.
- Tous les composants inclus dans ce kit sont destinés uniquement à ce test. Ne pas réutiliser ce test et/ou ses composants.
- Ce test doit être réalisé à température ambiante (15°C – 30°C) et dans un environnement sans humidité excessive.
- Ce test est à usage unique.
- Ce test doit être utilisé immédiatement après recueil de l'échantillon.
- Ne pas utiliser la cassette si elle est sortie de son emballage scellé depuis plus d'une heure.
- Ne pas utiliser la cassette si son emballage est endommagé.

PRÉLÈVEMENT

Rendez-vous sur notre page Youtube (Laboratoire Biosynex) en scannant le QR code suivant pour visualiser la vidéo de démonstration.

Avant sa réalisation, le test doit être mis à température ambiante pendant 2 heures. Il doit ensuite être utilisé entre 15 et 30°C. Veuillez lire la notice dans son intégralité. Munissez-vous d'une montre ou d'un chronomètre.

1

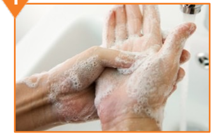

Lavez-vous les mains avant toute manipulation afin d'éviter une potentielle contamination manuportée.

2

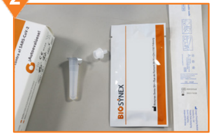

Ouvrez la boîte et sortez **tous les éléments**. Posez-les sur une surface plane et propre. Identifiez chaque élément du kit.

3

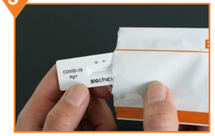

Déchirez l'encoche de la pochette. Sortez la cassette. **Utilisez le test dans l'heure.**

4

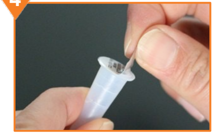

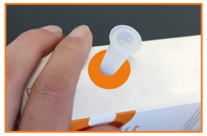

Enlevez l'opercule du tube contenant le diluant et posez-le dans le trou prédécoupé sur la boite.

5

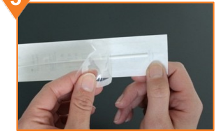

Sortir l'écouvillon de son emballage.

Prélèvement à réaliser dans chaque narine avec le même écouvillon :

6

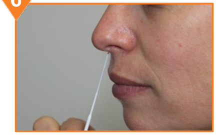

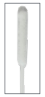

Insérez verticalement l'écouvillon dans une narine **jusqu'à ce que la résistance soit atteinte** (environ 3 cm).

7

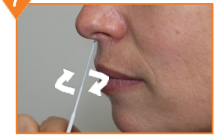

Faites rouler l'écouvillon **5 fois** le long de la muqueuse à l'intérieur de la narine pour vous assurer que le mucus et les cellules soient collectés. **L'écouvillon doit toucher les parois de la narine.**

8

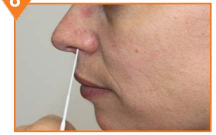

À l'aide du même écouvillon, **répétez ce processus dans l'autre narine**. Retirez l'écouvillon de la cavité nasale.

9

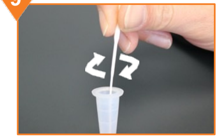

Insérez l'écouvillon dans le tube et tournez-le au moins **10 secondes** tout en appuyant l'extrémité contre le fond et le côté du tube d'extraction.

10

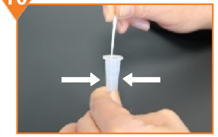

Extraire le maximum de liquide de l'écouvillon en « essorant » l'extrémité de l'écouvillon. Pour cela, appuyez plusieurs fois sur l'extérieur du tube. Retirez ensuite l'écouvillon et jetez-le.

RÉALISATION

11

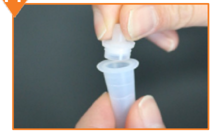

Mettez la tétine compte-goutte sur le tube.

12

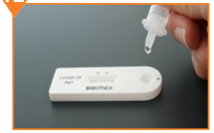

Déposez **doucement 4 gouttes** de solution dans le puits S.

13

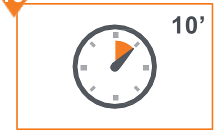

Déclenchez le chronomètre ou notez l'heure du dépôt. **Lire le résultat à 10 minutes.** Ne pas lire au-delà de 15 minutes.

Se référer à la partie « interprétation » pour la lecture du test.

INTERPRÉTATION

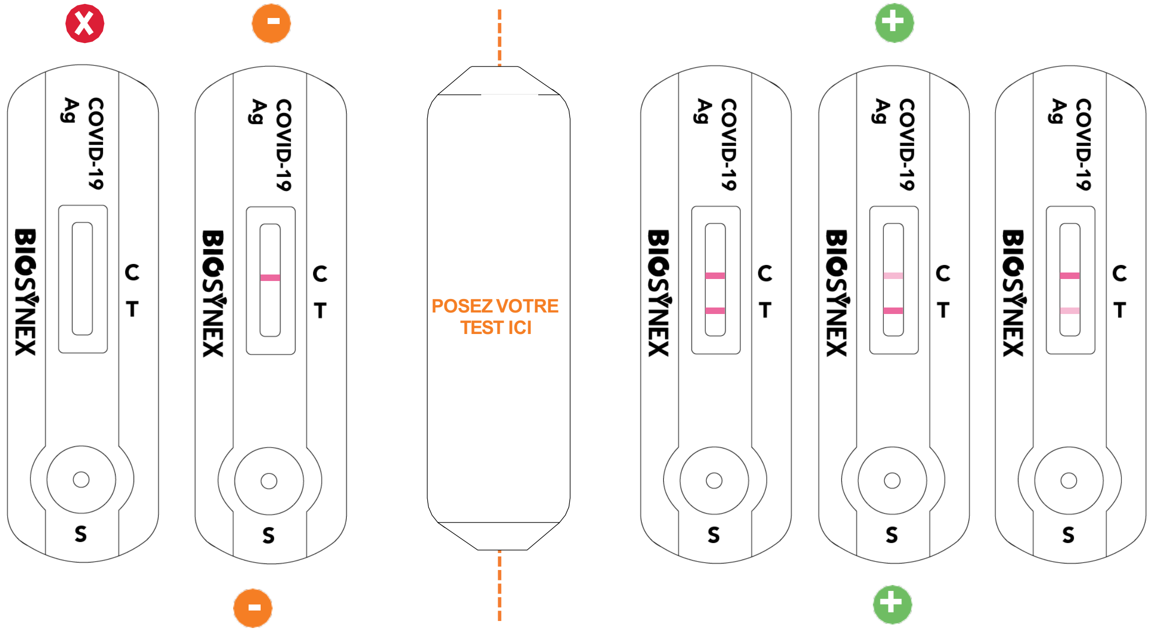

Le résultat du test est **NÉGATIF** si une bande colorée apparait uniquement dans la zone contrôle (C) et aucune bande colorée n'apparait dans la zone test (T) et ce, **quelle que soit l'intensité de la bande.**
Un résultat négatif indique l'absence d'antigènes du SARS-CoV-2.
Un résultat négatif n'exclut pas une infection au SARS CoV-2 récente.
Si vous pensez avoir été en contact avec le virus (avec une personne infectée) durant les derniers jours précédant la réalisation du test, nous vous invitons à refaire un test en laboratoire. Continuez de vous protéger et gardez une distanciation sociale.

Le résultat est **POSITIF** si 2 bandes colorées apparaissent sur la membrane. Une bande colorée apparait au niveau de la zone contrôle (C) et une au niveau de la zone test (T), **quelle que soit l'intensité des bandes**. Un résultat positif indique que vous êtes actuellement porteur du virus SARS-CoV-2.
Consultez votre médecin, continuez de vous protéger et gardez une distanciation sociale.

Si la ligne contrôle (C) n'apparait pas après avoir effectué le test, le résultat est considéré comme **INVALIDE**. Revoir la procédure et répéter le test avec une nouvelle cassette. Un volume insuffisant d'échantillon ou un échantillon trop visqueux, des techniques procédurales incorrectes (déchargement incorrect de l'écouvillon, conditions de température et d'humidité de réalisation du test) ou des tests ouverts depuis plus d'une heure voire périmés sont les raisons les plus vraisemblables de la non-apparition de la ligne de contrôle.

Pour éliminer votre test et ses composants, veuillez-vous référer aux règles en vigueur sur votre territoire.

INTERFÉRENCES

Aucune interférence positive ou négative n'a été démontrée avec les substances suivantes : Chlorhydrate d'ambroxol, antibiothérapeutique nasal (pommade à la mupirocine), furoate de mométasone en vaporisateur nasal, chlorhydrate d'oxymétazoline en vaporisateur, sirop antitussif Nin Jiom Pei Pa Kao, dipropionate de béclométhasone en aérosol nasal, solution orale de bromure de dextrométhorphane, acétonide de triamcinolone en vaporisateur nasal, solution orale de chlorhydrate de mucosolvane-ambroxol, Vaporisateur nasal au chlorhydrate d'azélastine, solution nettoyante nasale, NaCl, vaporisateur nasal au propionate, Hyland's 4 Kids Cold Cough Liquid Safe Natural Relief, vaporisateur nasal à l'eau de mer physiologique, Durham's Canker-Rid, gouttes oculaires à la tobramycine, bain de bouche à la listerine, sang total (4%), bain de bouche Scope, mucine.

PERFORMANCES

Sensibilité et spécificité
Le test Biosynex autotest antigénique COVID-19 Ag+ a été évalué à partir d'échantillons de patients. Un test moléculaire commercialisé a servi de méthode de référence. L'étude a porté sur 243 échantillons.

	%	95% CI
**Sensibilité**	96,0 %	90,9 - 98,7 %
**Spécificité**	99.2 %	95,4 - 99,9 %

Une étude de praticabilité a montré que :
- 90,9 % des différents types de résultats ont été interprétés correctement,
- 86,3 % des profanes ont réalisé le test sans avoir besoin d'aide.

LIMITES DU TEST

•Ce test ne permet pas de déterminer l'étiologie de l'infection respiratoire causée par d'autres micro-organismes que le SARS-CoV-2. Le test Biosynex autotest antigénique COVID-19 Ag+ est capable de détecter le SARS-CoV-2 viable et non-viable.
•Le test doit être utilisé pour la détection de l'antigène du SARS-CoV-2 à partir d'un prélèvement nasal. Ni la valeur quantitative ni le taux d'augmentation de la concentration du virus du SARS-CoV-2 ne peuvent être déterminés avec ce test qualitatif.
•La précision du test dépend de la qualité de l'échantillon de l'écouvillon. Des résultats faussement négatifs peuvent résulter d'un mauvais prélèvement..
• Le non-respect de la procédure de test peut affecter négativement la performance du test et/ou invalider le résultat du test.
•Si le résultat du test est négatif et que les symptômes cliniques persistent, il est recommandé de procéder à des tests supplémentaires en utilisant d'autres méthodes cliniques. Un résultat négatif n'exclut à aucun moment la présence d'antigènes du SARS-CoV-2 dans l'échantillon, car ils peuvent être présents en dessous du niveau de détection minimum du test ou si l'échantillon a été recueilli de manière incorrecte.
•Un résultat négatif n'exclut pas une infection par le SARS-CoV-2, en particulier chez les personnes qui ont été en contact avec le virus. Des tests de suivi avec un diagnostic moléculaire doivent être envisagés pour exclure l'infection chez ces personnes.
•Ce test ne se substitue pas à une consultation médicale ou au résultat d'une analyse biologique réalisée en laboratoire d'analyses médicales.
• Les résultats de test positifs n'écartent pas la possibilité de co-infections par d'autres pathogènes.
• Les résultats de test positifs ne permettent pas de différencier entre le SARS-CoV et le SARS-CoV-2.
•L'antigène détecté par le test est la protéine N. Les différentes variantes du virus décrites à ce jour dans certains pays (Royaume-Uni, Afrique du Sud, Brésil ...) concernent des mutations de la protéine Spike et n'ont donc pas d'impact sur la fonctionnalité du test.
• Des résultats erronés peuvent être obtenus :
- Si le test n'est pas utilisé selon les instructions de la notice,
- Si le sachet aluminium est endommagé ou si le test n'est pas réalisé immédiatement après l'ouverture de sachet aluminium,
-Si les conditions de conservation ne sont pas respectées ou si le test est réalisé après la date de péremption figurant sur le sachet aluminium.
•Il existe une possibilité d'obtenir des résultats faussement positifs notamment en cas de non-respect de la température à laquelle le test doit être effectué.
•Un résultat positif doit être impérativement confirmé par une analyse en laboratoire. Consultez votre médecin et ne pas prendre de décision médicale importante sans l'avis de votre médecin traitant.

Liste des tests autorisés par le ministère : https://covid-19.sante.gouv.fr/tests
Portail de signalement des événements sanitaires indésirables :
https://signalement.social-sante.gouv.fr/psig_ihm_utilisateurs/index.html#/accueil

Accessoire	Fabricant	Mandataire	Marquage CE
Écouvillon	**Jiangsu Changfeng Medical Industry Co.,** Ltd Touqiao Town, Guangling District, Yangzhou, Jiangsu 225109 China	**Llins Service & Consulting GmbH** Obere Seegasse 34/2, 69124, Heidelberg, Germany	CE0197
Ecouvillon	**Zhejiang Gongdong Medical Technology Co., Ltd.** No. 10 Beiyuan Ave., Huangyan 318020 Taizhou, Zhejiang, P.R.China	**Shangai International Holding Corp. GmbH (Europe)** Eiffestrasse 80, 20537 Halburg, Germany	CE0123

Attention, lire la notice d'utilisation
Nombre de tests par kit
LOT Numéro de lot
IVD Dispositif médical de diagnostic *in vitro*
Utiliser avant le
REF Référence
30°C 2°C Conserver entre 2-30°C
Fabricant
Ne pas utiliser si emballage endommagé
Dérogation de marquage CE
DIL Diluant
Ne pas réutiliser
Date de dernière révision : 04/2021
Signalétique commune TRIMAN
REF 859271
859272

Biosynex SWISS SA Rue de Romont 29-31
CH-1700 FRIBOURG - SUISSE
• client@Biosynex.com
• www.biosynex.com
• +33 3 88 78 85 24

Afin de nous aider, merci de prendre quelques minutes pour compléter le questionnaire disponible en flashant ce QR code ou via le lien suivant :
https://forms.gle/kRVdgTCaWdDzDwjQ7

IFU_859271_859272_FR_V05202104R01
